# Comparative effects of caffeine and paraxanthine on rowing performance and sleep quality: a randomized crossover study

**DOI:** 10.1080/15502783.2026.2650339

**Published:** 2026-03-31

**Authors:** Azize Bingol Diedhiou, Ulas Can Yildirim, Serhat Ozdenk, Dilara Erkan, Izzet Karakulak, Selin Yildirim Tuncer, Murat Turğut, Mehmet Can Gundem, Mehmet Veysi Bora, Fırat Akca

**Affiliations:** a Department of Coaching Education, School of Physical Education and Sports, Şırnak University, Şırnak, Türkiye; b Department of Coaching Education, Faculty of Sport Sciences, Sinop University, Sinop, Türkiye; c Department of Sport Management, Faculty of Sport Sciences, Sinop University, Sinop, Türkiye; d Department of Physical Education and Sports, Institute of Health Sciences, Ankara University, Ankara, Türkiye; e Department of Sport Management, Faculty of Sport Sciences, Mardin Artuklu University, Mardin, Türkiye; f Department of Coaching Education, Faculty of Sport Sciences, Lokman Hekim University, Ankara, Türkiye; g Department of Coaching Education, Faculty of Sport Sciences, Ankara University, Ankara, Türkiye

**Keywords:** Ergogenic aids, physical performance, time-trial performance

## Abstract

**Background:**

Although caffeine is widely used in athletes due to its ergogenic effects, the effects of its main metabolite, paraxanthine, on performance and sleep have not been adequately investigated.

**Purpose:**

This study aimed to comparatively investigate the effects of caffeine and its main metabolite paraxanthine on rowing performance and sleep quality.

**Methods:**

The study was designed as a randomized, double-blind, crossover study and included 14 male university-level rowers (21.6 ± 1.9 age; 2.2 ± 1 years of rowing experience). The participants participated in 2000-m rowing ergometer time trials under four different supplementation conditions (caffeine + paraxanthine, caffeine + placebo, paraxanthine + placebo, and placebo with 200  mg each). Performance data (completion time, mean power, and heart rate), sleep quality, and daytime sleepiness were assessed by subjective scales. The data were analyzed by repeated-measures analysis of variance (ANOVA) and Bonferroni post-hoc tests.

**Results:**

Compared with the placebo, the combined caffeine + paraxanthine condition was associated with faster 2000-m performance and higher mean power output (*p* = 0.044; Cohen's d = 0.30). Caffeine alone and paraxanthine alone did not show clear evidence of performance improvement in this sample, although estimates favored both conditions versus placebo. Conditions containing caffeine were associated with poorer subjective sleep quality, whereas paraxanthine alone showed more favorable sleep-related outcomes.

**Conclusion:**

These results indicate that the combined ingestion of caffeine and paraxanthine elicited the most pronounced performance benefits, while paraxanthine alone did not demonstrate clear standalone ergogenic efficacy at the administered dose. However, paraxanthine was associated with better subjective sleep outcomes compared to caffeine, suggesting that its potential value may be related more to tolerability rather than superior performance enhancement, particularly for athletes training in the evening. Study limitations, including the small sample size and lack of objective sleep measures, should be considered when interpreting the results.

**Practical applications:**

Paraxanthine may represent a practical option for athletes who prioritize sleep quality or experience sensitivity to caffeine-related sleep disturbances, although further dose‒response studies are required to clarify its ergogenic potential.

## 1. Introduction

Various nootropic nutritional supplements are preferred by athletes to improve their physical (exercise-related) and cognitive performance. Caffeine is one of the most commonly consumed supplements because of its stimulating effects [1]. The 2018 International Olympic Committee (IOC) reported that caffeine is among the five nutritional supplements whose positive effects on athletic performance are supported by scientific evidence [2]. It is known that caffeine use before exercise stimulates the central nervous system (CNS), which is its primary mechanism, improves cognitive functions [3], and reduces fatigue and drowsiness [4]. In addition to these effects that may enhance exercise and sport-specific performance, there are certain disadvantages resulting from genetic predispositions and excessive consumption.

Caffeine is metabolized by the CYP1A2 enzyme, which is found in the liver and is responsible for approximately 95% of the cytochrome P450 family [5]. Depending on the CYP1A2 gene polymorphism, enzyme activity may vary and lead to differences in metabolic rate between individuals [3]. These metabolic rates are important factors in determining both the level of benefit from the ergogenic effects of caffeine and the risk of exposure to potential side effects in athletes. Slow caffeine metabolizers are more sensitive to side effects than fast caffeine metabolizers [6].

Caffeine (1,3,7-trimethylxanthine) is metabolized in the liver to dimethylxanthines, ~70%–72% of which is paraxanthine. Metabolism occurs by the removal of a methyl group, resulting in the metabolites paraxanthine (1,7-dimethylxanthine), theobromine (3,7-dimethylxanthine), and theophylline (1,3-dimethylxanthine) [7]. Paraxanthine is the most predominant metabolite in humans and has higher plasma concentrations than other metabolites [8]. Paraxanthine has been proposed as a candidate option with caffeine-like nootropic effects and a potentially more favorable side-effect profile; however, evidence regarding its effects on physical performance in humans remains limited. In vitro, paraxanthine has been reported to exhibit adenosine receptor antagonism equal to or greater than caffeine [9].

Paraxanthine has a shorter plasma half-life compared to caffeine. It is eliminated more rapidly compared to caffeine and other metabolites. This elimination rate of paraxanthine minimizes possible side effects. It also acts as a more direct and safer stimulant by avoiding genetic differences in caffeine metabolism [10]. Scientific evidence supports that paraxanthine has a lower risk of toxicity [6], an anxiogenic effect [11], and cardiovascular and gastrointestinal side effects compared to caffeine [12]. Moreover, caffeine consumed during the day causes a decrease in the level of 6-sulfatoxymelatonin (the main metabolite of melatonin) the following night [13]. This is one of the mechanisms of sleep disruption. Especially when caffeine is consumed late at night, its stimulating effect continues due to its long half-life and may lead to insomnia [14]. Alford et al. [15] reported that, participants were given 4–8 mg/kg caffeine 20 min before bedtime. While 4  mg/kg of caffeine doubled the amount of time it took to fall asleep, 8  mg/kg of caffeine caused a decrease in sleep efficiency, deep sleep, REM sleep, and an increase in the number of awakenings. The short half-life of paraxanthine suggests that it will not affect sleep quality by ending its stimulant effect in a controlled manner.

The beneficial effects observed with caffeine use on endurance and sports performance have also been suggested for paraxanthine. In a study conducted by Jäger et al. [16] on mice, paraxanthine stood out as the most effective ingredient among various ergogenic aids in terms of muscle strength and aerobic endurance. Although current studies have supported the effects of paraxanthine on cognitive performance with human experiments [17,18], this indicates that further clinical research is needed, as there are not enough human-based studies in the literature regarding its effects on physical performance. Since rowing-specific human supplementation studies examining paraxanthine are scarce, the current study aims to address this gap by evaluating its effects on acute rowing exercise performance. Notably, in a placebo-controlled crossover study using a caffeine-containing supplement, plasma paraxanthine was elevated only under the timing condition in which a cycling time-trial performance benefit was observed, suggesting a potential role for paraxanthine that warrants direct investigation [19]. In the present study, performance refers specifically to acute rowing exercise performance assessed during a 2000-m indoor rowing ergometer time trial.

In addition to adenosine antagonism by paraxanthine that causes the same nootropic benefit as caffeine, another mechanism indicating that the benefits related to aerobic performance are through nitric oxide. Paraxanthine, in contrast to caffeine, theobromine, and theophylline, uniquely promotes nitric oxide neurotransmission. It increases blood flow due to the vasodilatory effect of nitric oxide. Thus, it can be associated with changes in exercise performance and cardiovascular health [20]. The aim of the current study was to examine and compare the effects of caffeine and paraxanthine on acute rowing exercise performance assessed during a 2000-m indoor rowing ergometer time trial in rowing athletes. The reason for conducting all the performance trials in this study in the evening was to evaluate both athletic output and related sleep effects at the same time.

## 2. Materials and methods

### 2.1. Participants

The investigation involved a group of 14 male university-level rowers, with an average age of 22 ± 2 years, a body mass of 77 ± 7 kg, a height of 183 ± 6 cm, a body fat percentage of 10.9 ± 4.1%, and a typical daily caffeine consumption of 335 ± 197  mg. These individuals had accrued an average of 2 ± 1 years of rowing training experience.

The required sample size was determined using G*Power software (version 3.1.9.4; Dusseldorf, Germany), based on an analysis of variance (ANOVA) design incorporating repeated measures and within-subjects factors. The calculation utilized an effect size (f) of 0.25, a significance level (alpha) of 0.05, and a statistical power of 0.90, assuming a within-subject correlation of *r* = 0.85. The model was specified for four conditions (within-subjects). The selected effect size and correlation assumption were based on prior repeated-measures time-trial performance studies using crossover designs and conservative (medium) planning assumptions. The calculation indicated that a minimum sample size of 12 participants was needed; therefore, 14 participants were recruited.

During their initial laboratory visit, participants underwent anthropometric assessments. Height and body mass were recorded using a Seca stadiometer (Seca Deutschland, Hamburg, Germany), while body fat percentage was evaluated via an InBody 770 body composition analyzer (InBody Co., Gangnam-Gu, Seoul, Korea). Following these measurements, the participants were provided with detailed study protocol information sheets and signed informed consent forms. The experimental methods were performed in accordance with the Declaration of Helsinki and approved by Sinop University, Human Research Ethics Committee (2025/93).

Eligibility for participation was restricted by specific exclusion criteria. Rowers were excluded from the study if they presented with:

(1) A medical condition that impaired their capacity to follow the study protocol,
(2) Current use of prescription medications,
(3) A confirmed allergy to corn flour,
(4) A diagnosed sleep disorder, or
(5) A physician's recommendation to limit or avoid caffeine intake.

### 2.2. Procedures

The research employed a double-blind, placebo-controlled, crossover design with randomization. The participants engaged in 2000-m indoor rowing time trials during four separate laboratory visits. Time trials were conducted under four primary testing conditions involving the intake of: a combination of caffeine and paraxanthine, caffeine paired with a placebo, paraxanthine combined with a placebo, or solely a placebo. All testing sessions were carried out in a manner that was both randomized and counterbalanced. An independent researcher generated the randomization schedule and allocated participants to the order of experimental conditions using an online randomization tool (Randomizer.org). The order was counterbalanced across participants, and all participants completed all conditions [21].

Every time trial was performed using a Concept II Model D Rowing ergometer (Concept II, Morrisville, VT, USA), with both the completion time and average power output (measured in watts) being documented. Additionally, heart rate measurements were taken during the tests via a Polar RS800CX instrument equipped with an H10 sensor (Polar Electro OY, Kempele, Finland). The testing sessions were spaced at least 72 h apart, with a maximum interval of 96 h, to guarantee adequate washout of treatments and sufficient recovery time.

To minimize the effect of circadian rhythms on performance, each participant performed their sessions at the same time of day (7:00 pm–8:00 pm). In addition to that, this time phase was specifically chosen (closer to the onset of sleep) for sessions to monitor the effects of different supplements on subjective night sleep quality and subsequent day wakefulness sensations. Upon the conclusion of each experimental trial, participants were asked to report any adverse effects they might have encountered. All participants were informed of the testing environment and protocol in advance, and procedures were applied consistently across sessions to ensure reliability.

### 2.3. 2000-m time trial

The 2000-m time trial was performed on the same rowing ergometer for each participant. The test-retest reliability of a 2000-m time trial on the Concept II rowing ergometer has been examined previously with well-trained rowers and was reported with a coefficient of variation (CV) of 0.6% [22]. However, reliability estimates may vary with training status; therefore, this value should be interpreted as a reference from well-trained cohorts rather than as a definitive estimate for the present sample. Each participant's warm-up routine was documented during the initial trial and then consistently reproduced in the subsequent trials. The time to complete the time trial was recorded. The stroke rate during the test was freely selectable by each subject, and the drag factor settings of the ergometers were adjusted to 140, as recommended by the Amateur Rowing Association for heavyweight men rowers [23]. Given the mean body mass of the present cohort (77 kg), this setting was considered appropriate for standardizing the testing conditions.

During the 2k test, after a self-selected warm-up, the athletes were required to row 2000 m in the least amount of time possible. This test is a standard criterion used for national team selection purposes in many countries [24,25] and was performed routinely by all the rowers in this study.

### 2.4. Supplementation protocol

Capsules, which are identical in appearance, were used to deliver the substances for all conditions. The same number of capsules (2) were administered 45 min before each trial to maintain blinding and ensure consistency. The supplementation conditions were as follows:

(1) 400 mg corn flour as a placebo (PLA)
(2) 200 mg caffeine + 200 mg paraxanthine (CAF+PAR)
(3) 200 mg caffeine + 200 mg placebo (CAF+PLA)
(4) 200 mg paraxanthine + 200 mg placebo (PAR+PLA)

### 2.5. Diet and caffeine consumption control

The research participants were instructed to abstain from alcohol consumption and vigorous physical training for a 24-h period preceding each experimental session. Throughout the duration of the study, the participants were advised to refrain from using any dietary supplements. All participants were required to maintain a detailed 24-h dietary record on the day prior to each testing session, as well as a weekly log of their caffeine intake. These food logs were qualitatively reviewed by the research team to ensure consistency in energy intake across test days, with basic macronutrient patterns (carbohydrates, protein, and fat) being compared session-to-session. To ensure consistency in energy consumption and hydration status, participants were directed to replicate their dietary intake, as documented in the food log, before every trial. Hydration was monitored informally by reminding participants to maintain regular fluid intake before and after each session and to report any noticeable changes in hydration behavior. Daily caffeine consumption was quantified using a modified version of the questionnaire developed by [26]. Additionally, a compiled list of common caffeine sources (coffee, tea, energy drinks, and chocolate) was provided to participants to assist accurate logging, and the caffeine content from various food and beverage sources was incorporated to determine the total daily caffeine intake. Based on this evaluation, all participants were classified as habitual moderate caffeine consumers, in accordance with the criteria established by [27]. To simulate conditions reflective of real-world athletic environments, as recommended by [28], participants were encouraged to maintain their usual daily caffeine intake throughout the study. This approach was implemented to mitigate the potential impact of caffeine withdrawal, as noted in previous literature [29].

### 2.6. Subjective sleep quality and daytime sleepiness measurements

Participants were directed to maintain their normal sleep patterns both prior to and following the experimental sessions. Each morning subsequent to a trial, they assessed their sleep quality utilizing a validated numeric rating scale, with scores ranging from 0 (indicating “best possible sleep”) to 10 (denoting “worst possible sleep”) [30]. Furthermore, levels of daytime sleepiness were evaluated in the afternoon through the application of the Karolinska Sleepiness Scale [31], which employs a numeric range from 1 (representing “extremely alert”) to 9 (signifying “very sleepy”).

### 2.7. Data analysis

The normality of the dataset was initially assessed using the Shapiro‒Wilk test. Following this, the sphericity assumption was evaluated with Mauchly's test, and the Greenhouse–Geisser correction was implemented whenever violations of sphericity were evident. To examine differences in test completion time, heart rate, power output, and subjective sleep parameters across the full duration of the testing period, a repeated measures analysis of variance (ANOVA) was employed. Where evidence of differences emerged, Bonferroni post hoc paired comparisons were conducted to identify specific differences between conditions. Effect sizes were quantified using Cohen's d for repeated measures, reported alongside their 95% confidence intervals (95% CI). These effect sizes were interpreted according to the following benchmarks: values less than 0.20 were deemed trivial, those ranging from 0.20 to 0.49 were classified as small, 0.50–0.79 were classified as moderate, and values of 0.80 or higher as large. Statistical computations were executed using SPSS software (version 30; IBM Corp., Armonk, New York, USA), while data visualizations were created with GraphPad PRISM software (version 10.4, GraphPad Inc., San Diego, CA, USA).

## 3. Results

**Figure 1.**
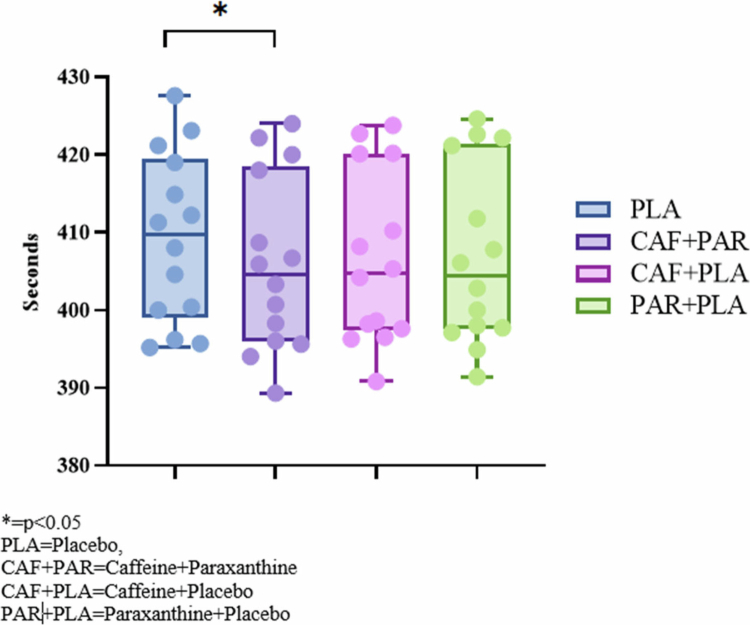
2000-m rowing ergometer time trial completion time.

The effects of various supplementations on the duration of rowing ergometer time trials are depicted in Figure 1. The rowing time-trial completion time differed across conditions (*p* = 0.006). Post-hoc analysis using the Bonferroni method indicated that the time trial was completed more rapidly in the CAF + PAR condition (405.91 ± 11.30 s) compared with the PLA condition (409.24 ± 10.92 s; *p* = 0.044; 95% CI: −0.046 to 0.640; d = 0.30). The CAF + PLA condition also showed a shorter mean completion time than PLA (406.62 ± 11.16 s; *p* = 0.087; d = 0.23), although the estimate remains uncertain based on the present sample.

**Figure 2.**
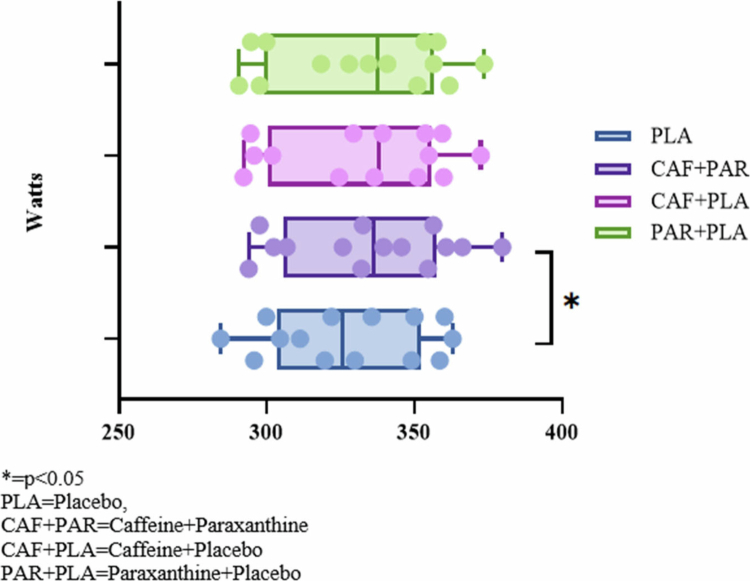
Mean power output during 2000 m rowing ergometer time trial.

**Figure 3.**
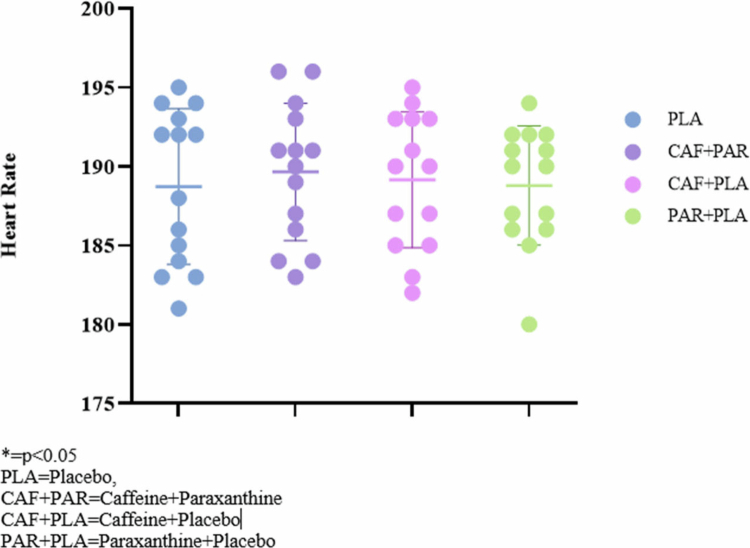
Average heart rate during 2000-m rowing ergometer time trial.

The average power output recorded during the time trial is presented in Figure 2. The mean power output differed across conditions (*p* = 0.013). Post-hoc analysis with the Bonferroni correction indicated higher mean power output in the CAF + PAR condition (335 ± 27 W) compared with PLA (327 ± 26 W; *p* = 0.046; 95% CI: −0.629 to 0.051; d = 0.31). The CAF + PLA condition also demonstrated higher mean power output than PLA (333 ± 28 W; *p* = 0.253; d = 0.22), although the magnitude of this difference remains uncertain (Figure 3).

The examination of heart rate data collected during time trials showed no notable differences across the conditions tested (*p* = 0.143).

**Figure 4.**
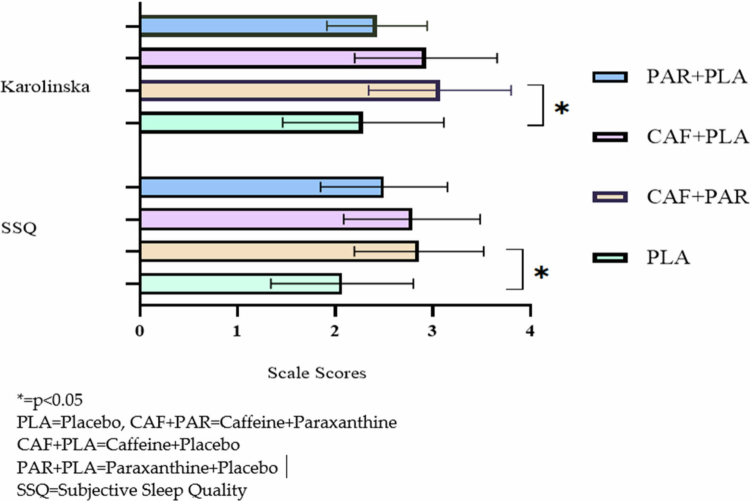
Subjective sleep quality and daytime sleepiness values.

Figure 4 presents data on subjective sleep quality and Karolinska Daytime Sleepiness Scale scores. The sleep-related outcomes differed across the conditions (*p* = 0.02). Compared with PLA, perceived sleep quality was lower in the CAF+PAR condition (*p* = 0.006; d = 1.13), and daytime sleepiness scores were higher (*p* = 0.006; d = 1.00). The CAF + PLA condition had a similar direction of effect on sleep quality (*p* = 0.071), although the estimate remains uncertain.

## 4. Discussion

The aim of this study was to comparatively examine the effects of caffeine and its main metabolite, paraxanthine, alone and in combination, on 2000-m rowing ergometer performance and subjective sleep parameters. The main findings showed that the combined caffeine and paraxanthine conditions were associated with faster 2000-m rowing performance (*p* = 0.044; d = 0.30). Although either compound alone showed favorable numerical trends, the estimates were uncertain in this sample (*p* = 0.087; d = 0.23). In contrast, subjective sleep quality tended to be poorer in conditions containing caffeine, whereas paraxanthine was associated with more favorable sleep-related outcomes.

The fact that CAF+PAR was associated with improved performance compared with placebo, while CAF and PAR alone did not show comparable effects, suggesting a potential complementary or synergistic interaction between these two compounds. However, this finding can be explained not only by true pharmacodynamic synergy but also by an increase in total paraxanthine exposure resulting from caffeine metabolism. Given that a significant portion of caffeine is metabolized to paraxanthine, the simultaneous presence of both endogenous and exogenous paraxanthine in circulation under the CAF+PAR condition may have led to an increase in the dose and duration of action of the active metabolite. Although both caffeine and paraxanthine act as non-selective antagonists of adenosine A1 and A2A receptors, they differ in their receptor binding tendencies, metabolic kinetics, and downstream neurophysiological effects [32]. Paraxanthine has been reported to bind to adenosine receptors with higher affinity, be cleared from the circulation more rapidly, and be associated with fewer anxiogenic effects and sleep disturbance compared to caffeine. The simultaneous activation of these different pharmacokinetic and pharmacodynamic properties may have resulted in more extensive or sustained central nervous system stimulation in the CAF+PAR combination compared to the other substances used alone. This could have positively influenced the regulation of motivation, motor unit involvement, or perceived effort during high-intensity exercise, contributing to the observed performance enhancement. However, since pharmacokinetic or mechanistic measurements related to plasma caffeine and paraxanthine levels were not performed in the present study, it is not possible to distinguish whether the observed performance enhancement was due to a true synergistic interaction or dose‒response relationship due to increased paraxanthine exposure. Therefore, this effect should be considered a hypothetical explanation, although biologically plausible based on the literature, and requires further verification.

A study conducted by Akça and Aras investigated the effects of various high-intensity interval training protocols on rowing performance improvements. The study revealed that an improvement of approximately six seconds corresponds to a gain of approximately two boat lengths in a single sculling race, which can be a decisive factor in determining the race outcome [33]. The 2024 Paris Olympics, as well as the 2024 World Championships, could serve as examples to illustrate this situation. For instance, in the men's single scull event at the 2024 Paris Olympics, the top three finishers recorded times of 6:37.57 (gold), 6:42.96 (silver), and 6:44.72 (bronze), respectively. Similarly, at the 2024 World Rowing Championships, the top three times were 6:49.68, 6:51.90, and 6:52.64 [34], respectively. Therefore, while the observed improvement may not reach clear statistical certainty, its potential relevance within the context of rowing ergometer performance should not be overlooked. However, previous studies have shown no direct correlation between indoor ergometer performance and rowing performance on water [35]. Thus, these findings should be interpreted carefully when applied to race performance.

Meanwhile, the mean power output during the time trial differed across conditions (*p* = 0.013). Specifically, this difference was observed between CAF+PAR and PLA (*p* = 0.046; d = 0.31). Our findings suggest that paraxanthine may contribute to performance outcomes when co-ingested with caffeine, whereas its effects in isolation are uncertain at the administered dose. Caffeine is a well-established ergogenic aid that has been a dominant focus in sports supplement research for several decades [36,37]. This prominence is due to its consistently demonstrated positive effects on performance across numerous studies [38–44]. Although the positive effects of paraxanthine on sport performance have not been widely reported in earlier studies, more recent research has begun to highlight its potential benefits [16,45]. In the present study, caffeine and paraxanthine administered alone showed favorable numerical trends in performance outcomes; however, the combined condition produced the most consistent performance-related outcomes. The *p*-values ranging from 0.05 to 0.10 suggest that responses to caffeine and paraxanthine may vary among individuals. Previous studies have noted the presence of individuals who respond to caffeine and those who do not, suggesting that this difference may stem from habitual caffeine consumption, genetic factors (e.g. CYP1A2 polymorphisms), and individual susceptibility to adenosine antagonism [46]. While this study did not aim to classify participants according to their response status, individual variability may partially explain the lack of clear effects in single-component conditions despite positive numerical trends.

However, the results indicate a different trend in terms of sleep quality. The participants in the CAF + PLA trial also reported poorer sleep quality compared to the PAR + PLA group. Although these differences are uncertain, a consistent pattern favoring paraxanthine was observed. These findings suggest that paraxanthine may offer a potential advantage in terms of parameters associated with improvement. The positive effects of caffeine on physical endurance and sports performance [45] have also been proposed for paraxanthine [16]. Caffeine is widely used by coaches and athletes as a performance-enhancing ergogenic aid and has various medical applications [47]. The stimulant effects of a non-selective antagonist of adenosine receptors (A1 and A2a), its stimulant effects are primarily attributed to the blockade of these receptors [48]. Additionally, high doses of caffeine may lead to adverse effects such as anxiety, tremors, headaches, and gastrointestinal irritation, which further restrict its therapeutic use [49]. Similar to caffeine, paraxanthine acts as a central nervous system stimulant. Both compounds share anti-adenosine properties; however, the existing literature consistently reports that paraxanthine exhibits slightly higher binding affinity for adenosine A1 and A2a receptors [50] while demonstrating lower toxicity and reduced anxiogenic effects compared to caffeine [11]. Similarly, compared to caffeine, paraxanthine is cleared from the blood more rapidly [10]. Even though numerous studies indicate that caffeine is often promoted for its effectiveness in mitigating fatigue, enhancing energy levels, supporting cognitive alertness [51], and modulating appetite [52], paradanthine may be preferred over caffeine because it has fewer adverse effects on the body. Furthermore, a dose‒response study demonstrated that paraxanthine exhibits nootropic effects even at acute doses as low as 50 mg [17].

For athletes engaging in evening training sessions, paraxanthine may serve as a more suitable ergogenic aid owing to its reduced impact on nocturnal [53] quality and next-day cognitive and physical alertness. When all the findings are considered simultaneously, it appears that the performance-enhancing advantage observed in the present study stems from the combined use of caffeine and paraxanthine, while paraxanthine alone may be more effective in situations where sleep quality and recovery are prioritized. This distinction has important practical implications for athletes to choose stimulants based not only on performance enhancement but also on their post-exercise recovery needs.

### 4.1. Limitations

The current study included only male, university-level athletes, which limits the generalizability of the findings to female athletes and other performance levels. Additionally, the evening hours of the tests made it difficult to fully control for the effects of circadian rhythms. The study was short-term in design, providing limited information on the long-term effects of supplements and adaptation processes. Additionally, no feedback was obtained from the participants regarding possible side effects. Side effect reports may provide predictive data on the positive effects of paraxanthine. The relatively small sample size (*n* = 14) may reduce the statistical power of the findings and limit generalizability. Objective sleep measures (e.g. actigraphy or polysomnography) were not employed, which restricts the scope of sleep quality assessment to subjective ratings. In addition, the single-item sleep quality scale (SQS) used in this study was originally psychometrically evaluated in clinical populations; although it provides a practical approach to capture within-subject changes across experimental conditions, it has not been formally validated in athlete populations, and findings should be interpreted accordingly. We also did not evaluate participants' caffeine tolerance or metabolizer status (e.g. via CYP1A2 genotyping), which may influence individual responses. Finally, all participants received fixed doses (200 mg) rather than weight-adjusted dosing. On a mg/kg basis, this corresponded to the lower end of the ergogenic range (mean ~2.6 mg/kg), which may have contributed to inter-individual variability and attenuated effects in the single-compound conditions. Additionally, the environmental conditions (e.g. room temperature, and humidity) were not strictly standardized, which is acknowledged as an additional limitation.

## 5. Conclusions

The present study suggests that combining caffeine and paraxanthine (CAF+PAR) may enhance rowing performance, reflected by faster 2000-m time-trial completion time and higher mean power output compared with placebo, whereas caffeine or paraxanthine alone showed no clear evidence of performance improvement in this sample. Notably, paraxanthine was associated with better sleep outcomes, in contrast to the sleep-disrupting effects of caffeine, suggesting a more favorable tolerability profile for athletes who train in the evening. However, the present findings do not support paraxanthine as a standalone ergogenic substitute for caffeine in terms of performance enhancement. These findings indicate that the primary performance benefit in this study was observed with the combined condition, warranting further research on different dosing strategies and the influence of genetic factors.

### 5.1. Practical implications

The data from this study reveal several practical applications important for future studies. From a performance perspective, the combined ingestion of caffeine + paraxanthine may be more effective than either compound alone in a competitive context. Paraxanthine alone may be considered when minimizing negative effects on sleep quality is a priority; however, its standalone ergogenic efficacy remains uncertain at the dose used in this study. Furthermore, considering that the combination of CAF+PAR provides performance improvements and that small differences may be important in a competitive context, this supplement can be used in pre-competition strategies. For athletes with caffeine sensitivity, paraxanthine offers a safer option with reduced side effects but should not be presented as providing similar ergogenic benefits to caffeine based on the current findings. Subsequent investigations should examine its dose-response relationship and long-term implications for sports performance and recuperation.

## Data Availability

The original contributions presented in the study are included in the article; further inquiries can be directed to the corresponding author.
